# Delayed Onset of a Daytime Nap Facilitates Retention of Declarative Memory

**DOI:** 10.1371/journal.pone.0012131

**Published:** 2010-08-12

**Authors:** Sara E. Alger, Hiuyan Lau, William Fishbein

**Affiliations:** Cognitive Neuroscience Subprogram, Department of Psychology, The City College of the City University of New York, New York, New York, United States of America; Harvard Medical School, United States of America

## Abstract

**Background:**

Learning followed by a period of sleep, even as little as a nap, promotes memory consolidation. It is now generally recognized that sleep facilitates the stabilization of information acquired prior to sleep. However, the temporal nature of the effect of sleep on retention of declarative memory is yet to be understood. We examined the impact of a delayed nap onset on the recognition of neutral pictorial stimuli with an added spatial component.

**Methodology/Principal Findings:**

Participants completed an initial study session involving 150 neutral pictures of people, places, and objects. Immediately following the picture presentation, participants were asked to make recognition judgments on a subset of “old”, previously seen, pictures versus intermixed “new” pictures. Participants were then divided into one of four groups who either took a 90-minute nap immediately, 2 hours, or 4 hours after learning, or remained awake for the duration of the experiment. 6 hours after initial learning, participants were again tested on the remaining “old” pictures, with “new” pictures intermixed.

**Conclusions/Significance:**

Interestingly, we found a stabilizing benefit of sleep on the memory trace reflected as a significant negative correlation between the average time elapsed before napping and decline in performance from test to retest (p = .001). We found a significant interaction between the groups and their performance from test to retest (p = .010), with the 4-hour delay group performing significantly better than both those who slept immediately and those who remained awake (p = .044, p = .010, respectively). Analysis of sleep data revealed a significant positive correlation between amount of slow wave sleep (SWS) achieved and length of the delay before sleep onset (p = .048). The findings add to the understanding of memory processing in humans, suggesting that factors such as waking processing and homeostatic increases in need for sleep over time modulate the importance of sleep to consolidation of neutral declarative memories.

## Introduction

There is a substantial body of evidence supporting the idea that sleep facilitates the consolidation of newly formed memories. Many behavioral studies demonstrate that a period of learning followed by sleep, as opposed to an equal time spent awake, benefits performance on a variety of tasks designed to measure different types of memories, such as implicit, procedural, or explicit, declarative memory [1]–[6]. Active processes occurring during sleep corresponding to different neurochemical states during each sleep stage aid in the strengthening of a new memory trace, stabilizing it and protecting it from interference [7], [8].

In the case of hippocampal-dependent declarative memories, non-rapid eye movement sleep (NREM, stages 1-4), specifically slow wave sleep (SWS, combined stages 3 & 4), is thought to facilitate the shift of the burden of memory reactivation from short term dependence on hippocampal areas to long term stores in the neocortex, a process known as systems consolidation [9], [10]. SWS is marked by large amplitude delta waves (.5–2 Hz), occupying progressively more of the brain wave pattern as the brain transitions from Stage 3 to Stage 4, as well as sharp-wave ripples, or fast hippocampal neural oscillations (140–200 Hz), grouped by the slow oscillations of SWS [11]. Neurons that were most recently fired during waking, such as those representing declarative memory, are thought to be reactivated by sharp-wave ripples and the theorized resulting long-term potentiation (LTP), the predominant candidate as a mechanistic explanation for synaptic consolidation within the network, gives rise to further strengthening between involved synapses due to structural modifications of the pre- and post-synaptic cells [12]–[14]. While a myriad of behavioral studies concluding that sleep facilitates memory consolidation [15]–[20], including the current experiment, cannot confirm this hypothesized mechanistic explanation, studies using imaging, tracking cerebral blood flow, and recording cellular firing activity during SWS elucidate the active role of sleep in memory processing [21]–[27].

While it is generally accepted that sleep facilitates greater recall for declarative information acquired prior to sleep, the temporal relationship between learning and the onset of sleep remains unclear. The majority of literature demonstrates that sleep must occur immediately after learning in order to benefit performance. However, a handful of recent studies find that sleep aids in the retention, or perhaps recovery, of memory even when it occurs after a substantial delay between learning and sleep onset [28], [29]. Performance on memory tests after delayed sleep has been shown in some cases to be similar or only slightly degraded from that seen when sleep immediately followed learning, with all sleep groups performing better than waking groups, implying an active role of sleep in memory processing rather than a passive, protective role. It is the timing of the benefits of sleep to memory that we address in the current study.

In order to control for circadian effects as well as hold constant the time of training and testing between all groups, we employed a nap design. In many sleep studies, researchers commonly use variations of one of two designs. In one, comparisons are conducted between overnight groups, those who sleep normally versus those who are deprived and remain awake, with results speaking more to the damaging effects of deprivation rather than the processing of memory over a sleep period. Alternatively, overnight sleep groups are compared to daytime wake groups, with the possibility of circadian confounds interfering with the results. Nap designs are increasingly used to practically address these issues and are of sufficient length to differentiate performance between sleep and wake groups [30]–[33]. Due to the length of an average nap when given a sleep opportunity of 90 minutes and the natural sleep architecture within this period, the predominance of NREM sleep aids in our examination of declarative memory processing.

The current study examined the time-dependent relationship between learning and sleep using a staggered nap schedule, in which separate groups napped starting either immediately, 2-hours, or 4-hours after a period of learning and were compared to a group that remained awake for the duration of the experimental manipulation. We employed a declarative visual recognition task, in which subjects viewed neutral pictures of people, objects, and landscapes and were later tested on their ability to distinguish previously viewed from new pictures. To both examine spatial memory as well as add complexity and richness to the memory trace, we also included a spatial aspect to the task, which required participants to view the picture stimuli in one of four quadrants on the computer screen and later recall where the picture had appeared. We hypothesized that a 90-minute nap, primarily comprised of NREM sleep, as compared to an equal period of wakefulness, would result in better performance at retest, compared to baseline measures, for both recognition as well as spatial memory. Based on differing evidence within the literature, we undertook the experiment with competing hypotheses with regard to the temporal relationship between sleep and performance. One hypothesis, supported by the majority of the literature and based on the idea that a window of time exists in which consolidation must occur [34], [35], predicted that as the delay between learning and sleep extended, the memory trace would degrade, resulting in a decrease in performance at retest. The opposing hypothesis, based on the idea that sleep can actively retain or recover memories [28], [29], [36], [37], predicted that all sleep groups would perform equally, and superior to the wake group, regardless of the length of the imposed delay.

## Methods

### Participants

Forty-three participants with an average age of 19.75 years (range 18–29) were recruited from the undergraduate population at the City College of the City University of New York. All subjects were reportedly in good health, free of sleep disorders or drugs that might impair or facilitate sleep, as determined by a screening interview. Participants were required to maintain a regular sleep schedule for the week prior to the experimental day, as verified by a subjective sleep log. Participants were also asked to refrain from alcohol or unnecessary drugs the day prior to as well as the day of the study, and caffeine the day of the study. Those who failed to meet these requirements were excluded prior to beginning the experiment. Of the original 43 participants, seven subjects were excluded from data analysis due to: inability to fall or remain asleep (3 nap participants), resulting in extended sleep latency and/or excessively fragmented sleep; inability to remain awake (1 wake participant); failure to correctly record responses on the answer sheet (1 participant); or below chance performance at test and retest (2 participants). The remaining 36 participants consisted of 17 males and 19 females. All participants signed informed consent. This study was approved by the City College of New York Institutional Review Board.

### Task

**Figure 1:**
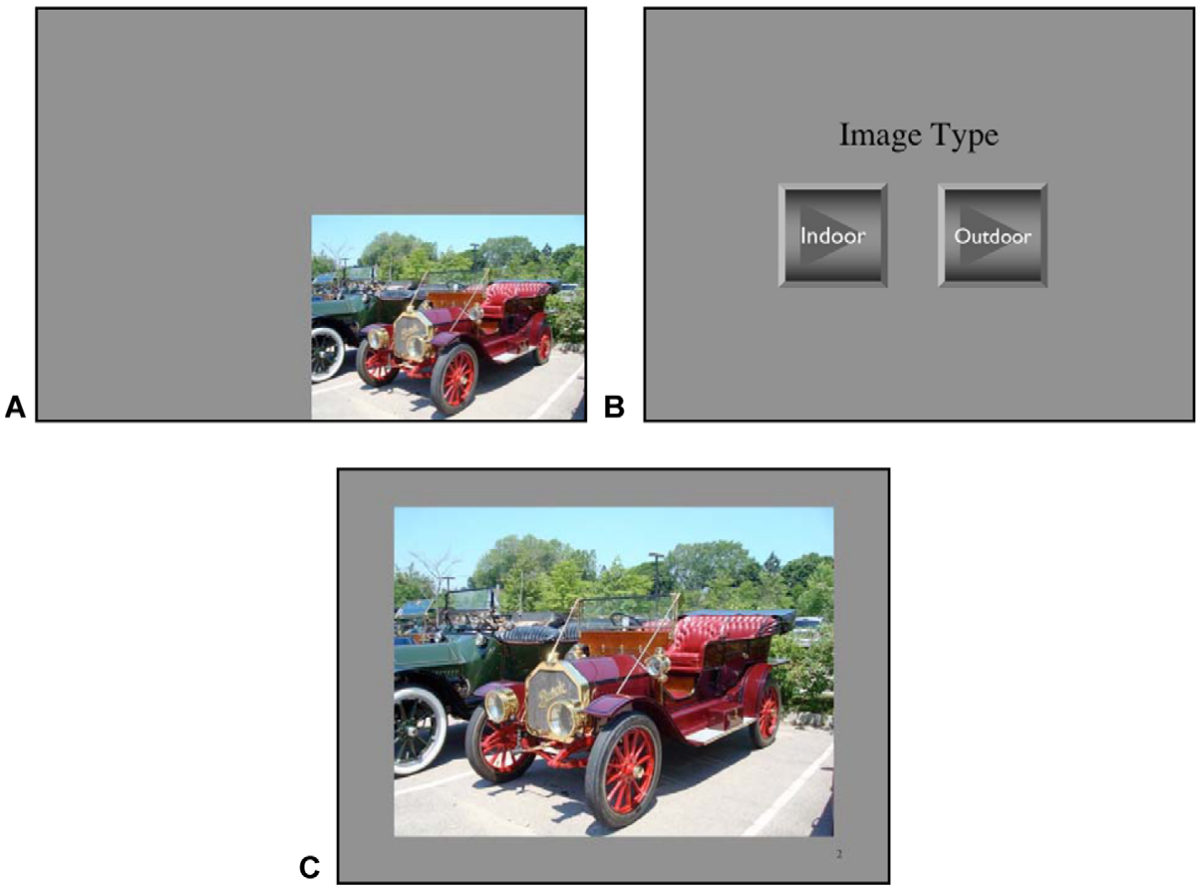
Declarative Visual Recognition Task Presentation. Declarative visual recognition task presentation: Each picture presentation began with a crosshair fixation screen, presented for 1 s to direct attention to the center of the screen. A) Individual neutral pictures were presented for 3 s in one of four quadrants of the computer screen. B) To ensure attention is being paid, participants were required to indicate whether the picture just viewed was an indoor or outdoor scene by clicking the correct button with the mouse, consequently advancing the slide show. C) During each testing session, pictures were presented mid-screen and participants were required to make “old/new” and spatial location decisions.

We used a visual recognition task in which 150 neutral pictures of non-renowned people, objects, and landscapes, matched for brightness and contrast, were presented to the participants via Microsoft PowerPoint on a 20″ computer screen. During the learning phase, participants viewed the pictures in five trials of thirty mutually exclusive pictures, counterbalanced across subjects and separated by two-minute inter-trial intervals. Trials began with a fixation crosshair for 1 s, followed by the target picture for 3 s, during which subjects simply viewed the stimulus. Each picture was presented in one quadrant of the computer screen, one per slide (**Figure 1A**), in order to add a spatial aspect to the task. Each picture slide was followed by a screen prompting the participant to respond via mouse click as to whether the previously picture was an indoor or outdoor scene (**Figure 1B**). This enabled confirmation of stimulus viewing, and offered a cogent behavioral marker to confirm that subjects paid attention to the stimuli. This decision was followed by another 1 s crosshair fixation before the next picture was presented.

Subjects were not asked to memorize the pictures. Rather, they were given three task objectives prior to the start of the trials. First, they were instructed to “take in” each picture as it was on the screen, noticing what the picture contained. Second, they were to note whether the scene was indoors/outdoors and told they would be asked to respond to this afterward. Finally, they were asked to take notice of which quadrant of the computer screen the picture appeared. Participants were not informed they would be tested on their memory of this task.

During two testing sessions, 100 new pictures of similar neutral people, objects, and landscapes were intermixed with previously viewed 150 pictures, 1/3 presented during initial baseline testing and 2/3 at retest. As each picture was presented centered on the computer screen (**Figure 1C**), participants were required to make an “old/new” decision, recorded on an answer sheet, as well as indicate which corner of the screen the picture had been presented if deemed as “old”.

### Procedures

At least one week prior to the experimental day, subjects were contacted via email to confirm their intent to participate, informed of prerequisites, and given the sleep log. On the day of the study, participants arrived at the Laboratory for Cognitive Neuroscience and Sleep at 10:00am, signed informed consent, and were introduced to the sound and light attenuated bedroom sleep chambers in order to facilitate adaptation to the surroundings. A brief description of the nature of the experiment was given, questions were answered, and participants completed the first Stanford Sleepiness Scale (SSS).

At 10:30am, nine electrodes were applied to all subjects in preparation for online standard polysomnograph recordings of electroencephalography (EEG; C3-A2, C4-A1), electro-oculography (EOG), and electromyography (EMG) using a five-channel polysomnographic montage in digital EEG acquisition software (Gamma System-Grass/Telefactor^tm^). In order to reduce experimental confounds, all participants were fitted with electrodes regardless of nap/no-nap grouping, and subjects were not informed of group assignment until after the learning phase.

At 11:00am, subjects were assigned to individual bedrooms for the remaining duration of the experiment. The learning phase then commenced in the bedrooms, with participants seated approximately 2′ from the computer monitor. After all 5 trials of pictures had been viewed, participants were immediately tested on a subset of the previously viewed pictures (50 pictures) intermixed with new, similar pictures (35 pictures). They were required to make a check mark on an answer sheet for each picture under either the “new” column or one of 4 “old” columns representing the four quadrants in which the “old” picture could have been presented, thus simultaneously measuring recognition as well as spatial memory. Subjects were allotted as much time as needed to complete this test.

**Figure 2:**
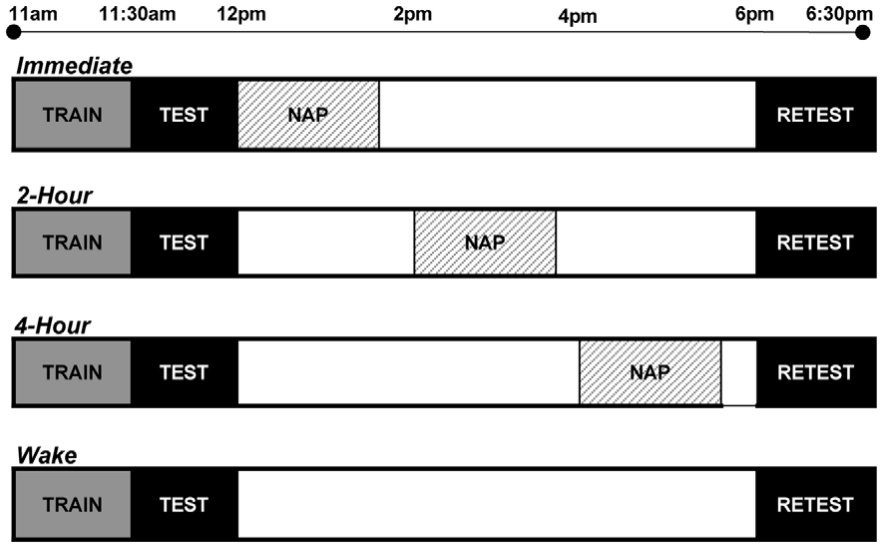
Description of Experimental Protocol. Experimental Protocol: Three experimental groups and the wake control group were all trained at 11am on the declarative memory recognition task, followed immediately by a testing session on a portion of the previously viewed stimuli intermixed with new pictures. After the test phase, sleep groups napped at staggered intervals, either immediately after testing at 12 noon, at 2pm, or at 4pm, while the control group remained awake. All groups were then retested at 6pm on the remaining stimuli, again intermixed with new pictures.

Following this initial testing phase, at approximately 12:00pm, participants were randomly assigned to one of four groups. Of three napping groups, one group immediately took a nap following testing. A second group remained awake until 2:00pm and then napped, while a third group remained awake until 4:00pm and then napped. The final, fourth group remained awake for the entire duration of the experiment (see **Figure 2** for Experimental Design). All nap subjects were given a 90-minute sleep opportunity, from the time of lights-out until lights-on. The subject either naturally awakened and remained awake if near the 90-minute mark, or was awakened from stage 1 or 2, as determined using the international criteria of Rechtschaffen and Kales [38], if the 90-minute mark was near and the subject could potentially progress into a deep stage of sleep before awakening naturally. Subjects were never awakened from SWS or REM sleep to reduce sleep inertia and the resulting disorientation and confusion experienced when emerging from these stages. While awake, subjects sat in a semi-recumbent position on the bed and passively watched light, animated comedies, chosen to reduce interference with viewed stimuli. They were allowed to eat and drink (non-caffeinated), but remained in the bedrooms aside from restroom breaks within the laboratory.

At 5:45pm, all subjects had electrodes removed and then sat in front of the computer for retesting. As before, participants completed another Stanford Sleepiness Scale and were tested on their recognition of the remaining previously viewed pictures (100 pictures) intermixed with new pictures (65 pictures), marking their answer sheets as described above, taking as much time as needed. Upon completion of this task, the subjects were debriefed on the purpose of the experiment and then allowed to leave.

## Results

Performance reflecting recognition and spatial memory was assessed as a within-subject repeated measure immediately after learning and again after the sleep/wake retention period (Test and Retest, respectively). Change in performance between these testing phases was compared between the different conditions (n = 9 per group), groups that napped at intervals (Immediate, 2-Hour, 4-Hour) as well as the control wake group (Wake). Recognition memory was measured as the percentage of correctly identified previously viewed “old” pictures, corrected for false alarm rate, for each test phase. Similarly, spatial memory was measured as the percentage of correctly identified picture locations of previously seen pictures for each test phase.

### Sleepiness Measures

The Stanford Sleepiness Scale uses a numerical scale 1–7 (1 being least sleepy, 7 most) to rate levels of alertness/sleepiness. Participants completed two SSS scales, upon arrival to the lab at approximately 10am and again immediately before retest at approximately 6pm. Group means ± SEM for each measure were, respectively; Wake  = 1.44±.176, 3.44±.294; Immediate  = 2.00±.289, 2.89±.351; 2-Hour  = 1.67±.236, 3.22±.324; 4-Hour  = 2.22±.401, 3.00±.333. There were no group differences using these subjective ratings at both the initial testing session (One-way ANOVA, F_3,32_ = 1.45, p = .248) as well at retest (One-way ANOVA, F_3,32_ = .570, p = .639).

### Sleep Data

We conducted one-way ANOVAs in order to compare sleep data between our three nap groups. No significant differences were found between the conditions for any specific sleep stage or characteristic. All groups had similar total sleep times (mean ± SEM, in min), with the Immediate group averaging 78.11±5.45, 2-Hour 74.94±8.46, and 4-Hour 76.44±6.90 (F_2,23_ = .051, p = .950). Sleep latency was also similar between groups; Immediate with 9.17±2.42, 2-Hour 6.12±1.45, and 4-Hour 7.2±2.00 (F_2,23_ = .566, p = .576). When examining the sleep stages, Stage 1 was omitted because it represents a brief transitional stage between wakefulness and sleep, and Stages 3 and 4 were combined in the conventional representation of SWS. Groups did not significantly differ in amount of Stage 2 sleep: Immediate 43.33±5.64, 2-Hour 34.75±4.97, and 4-Hour 38.28±3.20 (F_2,23_  = .829, p = .449); REM sleep: Immediate 15.22±6.23, 2-Hour 11.94±3.90, and 4-Hour 10.00±3.56 (F_2,23_ = .316, p = .732); or SWS: Immediate 9.67±3.63, 2-Hour 18.13±4.06, and 4-Hour 20.89±4.06 (F_2,23_ = 2.32, p = .121). However, when using independent t-tests to examine group differences in amount of SWS, we found a nearly significant difference between the Immediate and 4-Hour groups (t = −2.07, p = .055). Refer to **Table 1** for sleep data synopsis.

**Table 1:** Sleep Parameters (mean ± SEM, in minutes).

Condition	Total Sleep Time	S2	SWS	REM	Latency
Immediate	78.11±5.45	43.33±5.64	9.67±3.63	15.22±6.23	9.17±2.42
2-Hour	74.94±8.46	34.75±4.97	18.13±4.06	11.94±3.90	6.12±1.45
4-Hour	76.44±6.90	38.28±3.20	20.89±4.06	10.00±3.56	7.20±2.00
One-way ANOVA	F = .051, p = .950	F = .829, p = .449	F = 2.32, p = .121	F = .316, p = .732	F = .566, p = .576

### Spatial Memory

The percentages of correctly identified spatial locations for previously viewed stimuli were calculated for both the test and the retest sessions (correctly identified locations/total “old” pictures). Average scores for each group during the initial test for each group were as follows (mean %age ± SEM): Wake 32.67±3.00 percent, Immediate 30.78 ±3.65, 2-Hour 28.89±2.83, 4-Hour 32.36±5.58. During retest, average scores for each group were Wake 19.00±2.37 percent, Immediate 19.33±2.58, 2-Hour 21.22±1.96, and 4-Hour 22.47±4.38. Using repeated-measures ANOVA, where condition (Wake, Immediate, 2-Hour, 4-Hour) served as the between-subject factor, and time of testing (Test/Retest) served as the within-subject factor, we found a highly significant main effect of time (F_1,32_ = 43.30, p<.001), implying that, in all groups, performance on the spatial task deteriorated over time. We did not find a significant interaction between time of test and group (F_3,32_ = 1.15, p = .342).

### Recognition Memory

Averaging the percentage of correctly identified “old” pictures, corrected for bias by subtracting the percentage of false alarms for each subject, for the initial test phase (mean %age ± SEM) the control Wake group correctly recognized 76.27±2.52 percent of the old pictures, 80.29±3.35 for the Immediate group, 80.30±3.73 for the 2-Hour group, and 81.96±2.20 for the 4-Hour group. We confirmed that all participants performed similarly during the initial test session regardless of group, one-way ANOVA (F_3,32_ = .644, p = .593). At retest, the Wake group averaged a 59.84±3.68 percent, Immediate 61.53±4.84, 2-Hour 68.87±3.84, and 4-Hour 78.78±3.77 (**Table 2**).

**Table 2:** Percentage of correctly recognized previously viewed pictures at Test and Retest (mean ± SEM), with raw performance corrected for false alarms*.

Condition	Corrected Test Performance	Corrected Retest Performance
Wake	77.56–1.29 = 76.27±2.52	61.89–2.05 = 59.84±3.68
Immediate	82.22–1.93 = 80.29±3.35	63.22–1.69 = 61.53±4.84
2-Hour	82.22–1.92 = 80.30±3.73	69.89–1.02 = 68.87±3.84
4-Hour	85.78–3.82 = 81.96±2.22	79.78–1.00 = 78.78±3.77
One-way ANOVA	F = .644, p = .593	F = 4.51, p = .009
Repeated-Measures ANOVA	F = 3.19, p = .037	

*Reported in table as average raw performance percentage minus false alarm percentage.

**Figure 3:**
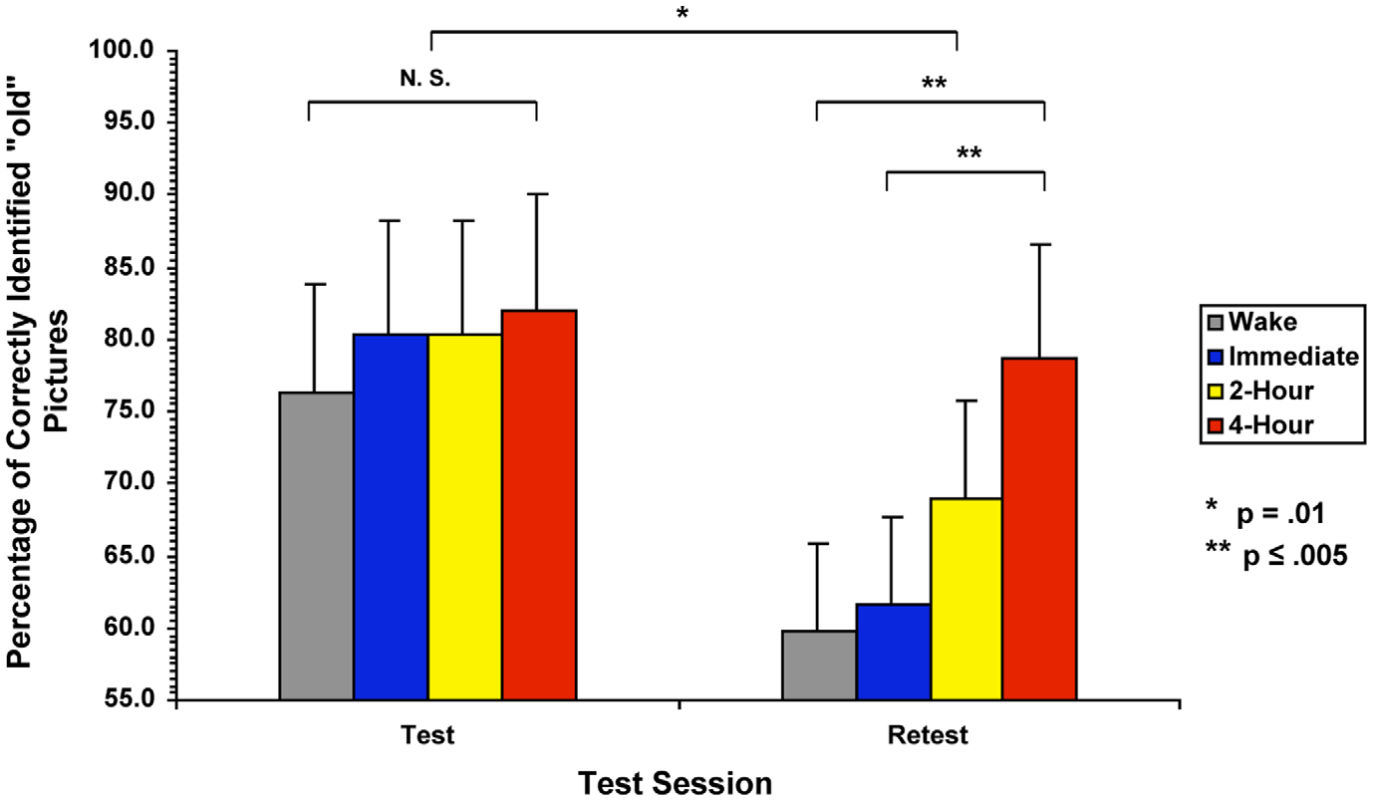
Corrected Performance on the Recognition Task at Test and Retest. Corrected performance at test and retest: The y-axis represents the percentage of correctly recognized previously viewed, or “old”, pictures, corrected for false alarms. The x-axis represents the scores for the control wake group (Wake) and the three experimental nap groups (Immediate, 2-Hour, and 4-Hour) for the Test and Retest sessions. At Test, all subjects performed similarly, with no significant differences found between the four groups. At Retest, the 4-Hour group performed significantly better than the Wake group (p = .002) and Immediate group (p = .005). Change in performance from test to retest was significant (p = .010), reflecting the interaction between group and test session.

Using repeated-measures ANOVA, we examined the change in recognition memory performance from test to retest. Condition (Wake, Immediate, 2-Hour, 4-Hour) served as the between-subject factor, while time of testing (Test/Retest) served as the within-subject factor. There was a highly significant overall main effect of time (F_1,32_ = 58.31 p<.001), indicating that, in all groups, performance on the recognition task deteriorated over time, from initial test until retest. We also found a significant interaction between time of testing and condition (F_3,32_ = 4.47, p = .010). Post-hoc analyses using Least Significant Difference (LSD) revealed that the 4-Hour delay group performed significantly better, with recognition memory deteriorating less than both the Wake and Immediate groups (p = .010, p = .044, respectively) (**Figure 3**).

**Figure 4:**
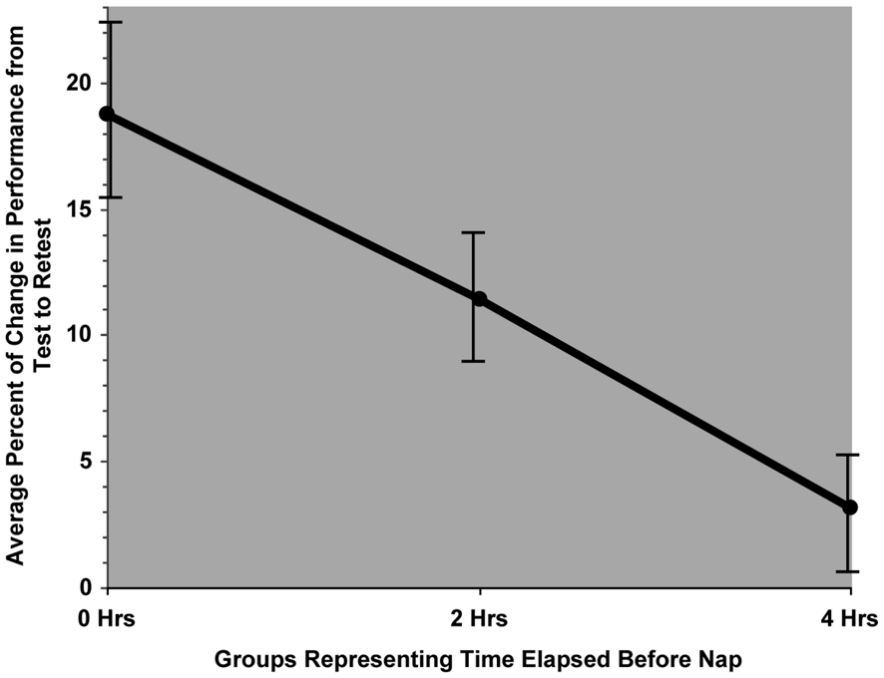
Spearman Correlation between Change in Performance from Test to Retest and Groups Representing Time Elapsed before Nap. Correlation between change in performance from test to retest and groups representing the average elapsed time before the nap: The y-axis represents the change in percentage of correctly recognized previously viewed, or “old”, pictures corrected for false alarms. The x-axis reflects the average amount of time elapsed before napping, at 0 hrs, 2 hrs, and 4 hrs post-learning, representing the Immediate, 2-Hour, and 4-hour groups' performance. Spearman's Rank Correlation revealed a significant negative correlation (p = .001), demonstrating that the 2-Hour group follows the trend of better performance with more time elapsed. Correlation was based on individual's change scores. Error bars indicate standard error of the mean.

Examining the nap groups using Spearman's Rank Correlation revealed a significant negative correlation between the individuals' change in performance from test to retest and the average elapsed time (0, 2, or 4 hours) between initial testing and sleep represented by the nap groups (r_s_ = −.623, p = .001). This correlation demonstrates that, while not significantly different from any particular group, the 2-Hour group falls in line with the progression of greater delay before sleep onset equaling better performance (**Figure 4**).

### Sleep Stages and Time Elapsed before Sleep

While we found no significant differences between the nap groups in the amount of total sleep time, sleep latency, Stage 2 sleep, REM sleep, or SWS, we noted that the amount of SWS appeared to increase as the length of the delay between learning and sleep extended. Spearman's Rank Correlation revealed a significant positive relationship to confirm this observation (r_s_ = .372, p = .048). Due to high levels of variance in the groups on both sleep and performance measures, no other correlations between sleep data and group performance were found.

## Discussion

We investigated the temporal relationship between learning and memory of a spatial and recognition task by using a staggered nap design with a waking control to assess the effects of delaying sleep onset. Out of harmony with some studies, we did not find a sleep benefit for spatial memory retention. However, after reevaluating our task, it may be reasonable to conclude that our spatial task requirement lacked enough precision to adequately test whether differences exist between sleep and an equivalent period wakefulness. While we cannot confirm this in the present study, recalling the visual location of a presented picture may not require the same spatial resources as moving through space, exploring one's environment, as do most animal studies through which our knowledge of sleep's contribution to spatial memory processing arise.

On the other hand, it appears that sleep does benefit declarative memory retention compared to an equal amount of time spent awake, although it is clear from the present results that the act of sleeping, alone, is not enough to account for the differences in retention. In the present experiment, we entertained two possible hypotheses regarding the role of sleep in memory retention, and anticipated that the memory trace would either degrade as the length of the delay between learning and sleep increased (consistent with classical interference theory), or that sleep would actively sustain memory retention regardless of the delay, resulting in equal performance among sleep groups, (consistent with systems consolidation theory). However, we found neither. Quite the opposite, better performance on the recognition task was seen the longer participants remained awake before nap onset.

As stated before, subjects were not asked to memorize the pictures and were not informed that they would be tested on their recognition of previously seen pictures. We cannot rule out that once the initial test was given, subjects may have anticipated an additional test later in the day. However, this is unlikely to have affected consolidation or subsequent recognition. Once subjects realized they were to be tested on the material, they had already completed viewing the stimuli, so anticipation of a test did not bias their encoding of the material. The stimuli presented in the two testing sessions were mutually exclusive so that subjects were not re-exposed to any previously viewed stimuli during the first test that would aid them in retest performance. On the off chance that a subject did anticipate a future test and could rehearse the remaining pictures in his/her mind's eye, we attempted to reduce practice effects by exposing the subjects to a visual passive activity, watching animation, for the duration of the delay not spent sleeping. While we did not administer subjective measure to determine whether or not rehearsal was taking place, we would anticipate that the occurrence of rehearsal would be evenly spread across the groups and not contribute to the group-specific differences in performance we found from test to retest.

We focus on what we see as several possible considerations in our study that lend explanation to this unanticipated finding.

### 

#### Neutral vs. Emotional Stimuli Processing during Wake

One possible explanation for the results centers on our task. The declarative visual recognition task consisted of only *neutral* pictures of people, objects, and landscapes. Subjects were asked to view the stimuli with no accompanying narrative or emphasis on the importance of remembering the picture. Reviewing the literature using recognition tasks comparing neutral and emotional stimuli, the majority of studies found benefits to performance after a period of sleep only when the stimuli contained an *emotional* component, making the memory trace stronger and more salient, with corresponding stronger neuronal connections [39]–[43]. In these studies, employing both emotional and neutral stimuli in a sleep/no sleep design, sleep provided no additional benefit, compared to remaining awake, for performance on neutral items, unlike for emotional items. However, these studies used only one sleep period, usually occurring immediately after learning, in contrast to the delayed sleep paradigm we employed. To this extent, our results are compatible with the literature, with no performance benefit found in our Immediate nap group.

Since increasing the delay between learning and sleep resulted in significantly better performance from learning to retention test, we propose that a period of time spent awake, further processing the neutral memories, strengthens the memory trace and makes it more salient, possibly through time-dependent consolidation occurring during a time of passive activities. It is only then, when the memories are strengthened, that sleep can play a role in facilitating the retention of the memory. It is also possible that perhaps distinct aspects of the memory trace are consolidated differentially during wake and sleep, resulting in combined better performance. Such dissociated improvement has been demonstrated, although in procedural memory, providing evidence for different roles for wake and sleep in memory processing [44]. While these theories could be plausible, further research, ideally using imaging techniques, would be necessary to confirm reactivation of the memory trace during this resting wake period.

It is also possible that we have uncovered a memory-dependent sleep window for human declarative memory, similar to the paradoxical sleep windows described by Smith [34], [45]. He describes these windows as post-training periods of time in which REM sleep is critical and has been augmented by learning, increasing the amount and length of REM sleep. These critical windows generally occurred several hours, rather than immediately, after rodent mass training and only when sleep occurred in this period did performance benefit. The fact that we found a period of augmented SWS that benefited performance several hours after training is an intriguing parallel with Smith's work, but clearly the evidence to support this claim in humans is weak at this stage.

#### Circadian Rhythmicity

While our use of a daytime nap was intended to diminish the effects of circadian differences between groups in terms of time of learning and testing, held constant in the current study, the delayed nap design did not fully eliminate all possible circadian confounds. In one overnight study, Plihal and Born explored the differences in declarative and procedural memory processing by different sleep stages by taking advantage of the natural circadian structural differences between SWS and REM sleep, with greater amounts of SWS occurring in the early night and lessening toward morning, while REM sleep increases toward late morning. This homeostatic exchange in amounts of REM and SWS continues during the day, so that a 1pm nap theoretically contains equal amounts of REM sleep and SWS, while a 5pm nap might show more SWS than REM sleep. The present data lends support to this idea. We found that the ratio of SWS to REM sleep reflects this circadian shift, with the amount of SWS increasing as the delay between learning and sleep increased. At the same time, the amount of REM sleep reciprocally decreased over this time period. This increase in SWS over time is thought to be mediated by increased adenosine release and accumulation over extended waking periods, resulting in greater slow-wave activity at sleep-onset [46]–[49]. It should be noted though that circadian differences found in this study could only be used to explain performance differences as it specifically applies to the type of sleep each group predominantly achieved, since time of learning and testing was consistent between the groups.

When examining our unusual results in the context of homeostatic changes in sleep over time, one could conclude that the most logical explanation for the performances difference we found between the groups may be due to the later nap groups having the lowest level of homeostatic sleep pressure at the time of *retesting*. However, if post-sleep-increased sleep pressure at retest was the cause of our results, then the immediate nap group, whose homeostatic sleep pressure would have been greatly reduced with their noon nap, should be performing far better than the wake group, whose pressure had been building from the time they awakened in the morning until the 6pm retest. We see, in fact, that this is not the case.

#### Homeostatic Need for SWS

Greater amounts of SWS seen in the 2-Hour and 4-Hour delay groups compared to the Immediate group may not strictly be due to circadian differences in naptime, but to an increase in the homeostatic need for SWS built up *prior to* the nap. According to Tononi & Cirelli's Synaptic Homeostasis Hypothesis [50]–[52], one function of sleep, specifically SWS, is to downscale, or decrease, synaptic weights. Over a period of wakefulness and with the acquisition of new information, synapses are continually potentiated and synaptic resources are consumed, reaching an eventual saturation point after which the increased threshold for potentiation prevents further consolidation of new information. This increased potentiation is correlated with the homeostatic regulation by SWS, which restores synaptic resources and lowers the threshold. Performance on memory tasks for information learned prior to sleep benefits from this downscaling, with weak synaptic connections related to everyday “noise” falling below threshold, while synaptic connections related to learned material, or the “signal”, remaining above threshold, increasing the ratio of signal to noise. The more information that is acquired prior to sleep, be it learned information or simple sensory experiences over everyday life, the greater the homeostatic need for SWS and, consequently, more slow wave activity is seen after sleep onset. Whether the increase in amount of SWS we observed is due to this homeostatic increase due to learning alone or in conjunction with circadian rhythmicity, Tononi and Cirelli's theory of the function of SWS lends an elegant mechanistic model for our results.

We must emphasize that the hypothesized synaptic homeostasis theory is not mutually exclusive from, and may occur in conjunction with, the active systems consolidation theory discussed earlier in this paper, which gives an active role to slow-wave activity in the reactivation-based shift of new memories from short term to long term stores, resulting in better, more stabilized performance after sleep compared to wake. Given the differences between the groups in amount of SWS attained, this systems consolidation theory can also be used to account for our unanticipated behavioral data. We focused on the Synaptic Homeostasis Hypothesis in our discussion because it also explains the increases in SWS seen in our groups after learning and subsequent time spent awake as well as lending explanation as to how slow-wave activity benefits performance.

#### Directions for Future Research

With these unexpected results, new directions for future research present themselves in order to clarify the present findings and possibly provide support to potential mechanistic explanations for the current results. To all of the following possible protocols, we would also add a psychomotor vigilance test of general cognitive ability in order to aid in differentiating changes in performance due to consolidation effects from possible blanketing changes in ability to complete general cognitive tasks due to potential confounding homeostatic differences between groups.

First, of interest would be a follow-up study comparing performance using both emotional and neutral stimuli, employing the current protocol, in order to examine whether or not the neutrality of our recognition memory task contributed to our findings. Varying the salience of the viewed stimuli, perhaps without the spatial aspect in order to simplify the comparisons, would allow a clearer picture of the benefit of sleep for different types of recognition memory.

Another possible area of interest is the extent of the delay time between learning and sleep onset. In order to more clearly map the time-dependent benefit of sleep on memory, the delay could potentially be extended over a greater period of time, while still holding the time of learning and testing constant, with more napping groups introduced in the interim.

Finally, focusing on SWS as discussed at length both in regard to sleep-dependent systems consolidation theory as well as the Synaptic Homeostasis Theory, a follow-up study in which amount of SWS is somehow held constant between all nap groups over the delay may aid in understanding the contribution of this type of sleep to declarative recognition memory processing. While we cannot say with certainty, it is a possibility that had all nap groups achieved equal amounts of SWS, we might have found support for our hypothesis that sleep actively retains the memory trace, with equal performance across nap groups, regardless of the length of the delay. One method of possibly controlling for amount of SWS as well as homeostatic confounds would be to hold constant the time at which all groups napped and were subsequently retested, but stagger the pre-nap times of training and initial testing, so that, similar to this study, groups would learn 4 hrs, 2 hrs, or immediately prior to napping. This would eliminate homeostatic sleep differences, although groups would be learning at different times of the day, potentially confounding in itself. There are inherent confounds with manipulating the natural amount of SWS achieved in a nap as well, either through truncating the length of the nap or inducing slow waves to boost slow wave activity in the earlier nap groups, but the idea of exploring this area in future projects if proper controls can be achieved is appealing and would certainly add to our understanding of the role for SWS in memory processing.

## References

[1] Fishbein W, Gutwein BM (1977). Paradoxical sleep and memory storage processes.. Behavioral Biology.

[2] Plihal W, Born J (1997). Effects of early and late nocturnal sleep on declarative and procedural memory.. Journal of Cognitive Neuroscience.

[3] Smith CT (2001). Sleep states and memory processes in humans: procedural versus declarative memory systems.. Sleep Medicine Reviews.

[4] Walker M, Stickgold R (2004). Sleep-dependent learning and memory consolidation.. Neuron.

[5] Stickgold R (2005). Sleep dependant memory consolidation.. Nature.

[6] Born J, Rasch B, Gais S (2006). Sleep to remember.. Neuroscientist.

[7] Hasselmo ME (1999). Neuromodulation: Acetylcholine and memory consolidation.. Trends in Cognitive Sciences.

[8] Ellenbogen JM, Payne JD, Stickgold R (2006). The role of sleep in declarative memory consolidation: passive, permissive, active or none?. Current Opinion in Neurobiology.

[9] Buzsaki G (1989). Two-stage model of memory trace formation: A role for “noisy” brain states.. Neuroscience.

[10] Buzsaki G, Leung LW, Vanderwolf CH (1983). Cellular bases of hippocampal EEG in the behaving rat.. Brain Research.

[11] Battaglia FP, Sutherland GR, McNaughton BL (2004). Hippocampal sharp wave bursts coincide with neocortical “up-state” transitions.. Learning & Memory.

[12] Diba K, Buzsaki G (2007). Forward and reverse hippocampal place-cell sequences during ripples.. Nature Neuroscience.

[13] Sirota A, Csicvari J, Buhl D, Buzsaki G (2003). Communication between neocortex and hippocampus during sleep in rodents.. PNAS.

[14] Behrens CJ, van den Boom LP, de Hoz L, Friedman A, Heinemann U (2005). Induction of sharp-wave ripple complexes in vitro and reorganization of hippocampal networks.. Nature Neuroscience.

[15] Ekstrand BR (1967). Effect of sleep on memory.. Journal of Experimental Psychology.

[16] Ellenbogen JM, Hulbert JC, Stickgold R, Dinges DF, Thompson-Schill, SL (2006). Interfering with theories of sleep and memory: Sleep, declarative memory, and associative interference.. Current Biology.

[17] Gais S, Molle M, Helms K, Born J (2002). Learning-dependent increases in sleep spindle density.. The Journal of Neuroscience.

[18] Gais S, Born J (2004). Declarative memory consolidation: Mechanisms acting during human sleep.. Learning & Memory.

[19] Kali S, Dayan P (2004). Off-line replay maintains declarative memories in a model of hippocampal-neocortical interactions.. Nature Neuroscience.

[20] Rasch B, Buchel C, Gais S, Born J (2007). Odor cues during slow-wave sleep prompts declarative memory consolidation.. Science.

[21] Wilson MA, McNaughton BL (1994). Reactivation of hippocampal ensemble memories during sleep.. Science.

[22] Louie K, Wilson MA (2001). Temporally structured replay of awake hippocampal ensemble activity during rapid eye movement sleep.. Neuron.

[23] Ji D, Wilson MA (2007). Coordinated memory replay in the visual cortex and hippocampus during sleep.. Nature Neuroscience.

[24] Molle M, Marshall L, Gais S, Born J (2004). Learning increases human electroencephalographic coherence during subsequent slow sleep oscillations.. PNAS.

[25] Peigneux P, Laureys S, Fuchs S, Collette F, Perrin F (2004). Are spatial memories strengthened in the human hippocampus during slow-wave sleep?. Neuron.

[26] Takashima A, Petersson KM, Rutters F, Tendolkar I, Jensen O (2006). Declarative memory consolidation in humans: a prospective functional magnetic resonance imaging study.. PNAS.

[27] Gais S, Albouy G, Boly M, Dang-Vu TT, Darsaud A (2007). Sleep transforms the cerebral trace of declarative memories.. PNAS.

[28] Fenn KM, Nusbaum HC, Margoliash D (2003). Consolidation during sleep of perceptual learning of spoken language.. Nature.

[29] Backhaus J, Hoeckesfeld R, Born J, Hohagen F, Junghanns K (2007). Immediate as well as delayed post learning sleep but not wake enhances declarative memory consolidation in children.. Neurobiology of Learning and Memory.

[30] Mednick SC, Nakayama K, Stickgold R (2003). Sleep-dependent learning: A nap is as good as a night.. Nature Neuroscience.

[31] Schabus M, Hodlmoser K, Pecherstorfer T, Klosch G (2005). Influence of midday naps on declarative memory performance and motivation.. Somnologie.

[32] Backhaus J, Junghanns K (2006). Daytime naps improve procedural memory.. Sleep Medicine.

[33] Tucker MA, Hirota Y, Wamsley EJ, Lau H, Chaklader A, Fishbein W (2006). A daytime nap containing solely non-REM sleep enhances declarative but not procedural memory.. Neurobiology of Learning and Memory.

[34] Smith CT, Tenn C, Annett R (1991). Some biochemical and behavioural aspects of the paradoxical sleep window.. Canadian Journal of Psychology.

[35] Fishbein W, McGaugh JL, Swarz JR (1971). Retrograde amnesia: Electroconvulsive shock effects after termination of rapid eye movement sleep deprivation.. Science.

[36] Racsmany M, Conway MA, Demeter G (2010). Consolidation of episodic memories during sleep: Long term effects of retrieval practice.. Psychological Science.

[37] Baran B, Wilson J, Spencer RMC (2010). REM-dependent repair of competitive memory suppression.. Experimental Brain Research.

[38] Rechtschaffen A, Kales A (1968). A manual of standardized terminology, techniques and scoring system for sleep stages in human subjects..

[39] Hu P, Stylos-Allan M, Walker MP (2006). Sleep facilitates consolidation of emotional declarative memory.. Psychological Science.

[40] Payne JD, Stickgold R, Swanberg K, Kensinger EA (2008). Sleep preferentially enhances memory for emotional components of scenes.. Psychological Science.

[41] Ritchey M, Dolcos F, Cabeza R (2008). Role of amygdala connectivity in the persistence of emotional memories over time: An event related fMRI investigation.. Cerebral Cortex.

[42] Nishida M, Pearsall J, Buckner RL, Walker MP (2009). REM sleep, prefrontal theta, and the consolidation of human emotional memory.. Cerebral Cortex.

[43] Sterpenich V, Albouy G, Darsaud A, Schmidt C, Vandewalle G (2009). Sleep promotes the neural reorganization of remote emotional memories.. The Journal of Neuroscience.

[44] Cohen DA, Pascual-Leon A, Press DZ, Robertson EM (2005). Off-line learning of motor skill memory: A double dissociation of goal and movement.. PNAS.

[45] Smith C (1996). Sleep states, memory processes and synaptic plasticity.. Behavioural Brain Research.

[46] Radulovacki M, Miletich RS, Green RD (1982). N^6^ (L-Phenylisopropyl) adenosine (L-PIA) increases in slow-wave sleep (S_2_) and decreases in wakefulness in rats.. Brain Research.

[47] Benington JH, Kodali SK, Heller HC (1995). Stimulation of A_1_ adenosine receptors mimics the electroencephalographic effects of sleep depreivation.. Brain Research.

[48] Porkka-Heiskanen T, Strecker RE, Thakkar M, Bjorkum AA, Greene RW, McCarley RW (1997). Adenosine: A mediator of the sleep-inducing effects of prolonged wakefulness.. Science.

[49] Retey JV, Adam M, Honegger E, Khatami R, Luhmann UFO (2005). A functional genetic variation of adenosine affects the duration and intensity of deep sleep in humans.. PNAS.

[50] Tononi G, Cirelli C (2003). Sleep and synaptic homeostasis: a hypothesis.. Brain Research Bulletin.

[51] Tononi G, Cirelli C (2006). Sleep function and synaptic homeostasis.. Sleep Medicine Review.

[52] Huber R, Ghilardi MF, Massimini M, Tononi G (2004). Local sleep and learning.. Nature.

